# Maternal Blood Lipid Profile during Pregnancy and Associations with Child Adiposity: Findings from the ROLO Study

**DOI:** 10.1371/journal.pone.0161206

**Published:** 2016-08-25

**Authors:** Aisling A. Geraghty, Goiuri Alberdi, Elizabeth J. O’Sullivan, Eileen C. O’Brien, Brenda Crosbie, Patrick J. Twomey, Fionnuala M. McAuliffe

**Affiliations:** 1 UCD Obstetrics and Gynaecology, School of Medicine, University College Dublin, National Maternity Hospital, Dublin 2, Ireland; 2 Clinical Chemistry, St. Vincent’s University Hospital, Dublin 4, Ireland; 3 UCD School of Medicine, University College Dublin, Dublin, Ireland; Universite de Montreal, CANADA

## Abstract

**Background:**

The *in-utero* environment affects fetal development; it is vital to understand how maternal diet during pregnancy influences childhood body composition. While research indicates that triglycerides in hyperglycaemic women may increase birth weight, little is known about this relationship in euglycemic women. This study examines the relationship between maternal blood lipid status and infant adiposity up to 2 years of age.

**Methods:**

Data from 331 mother-child pairs from the ROLO longitudinal birth cohort study was analysed. Maternal dietary intakes were recorded and fasting blood lipids, leptin and HOMA were measured in early and late pregnancy and cord blood. Infant anthropometric measurements and skin-fold thicknesses were recorded at birth, 6 months and 2 years. Correlation and regression analyses were used to explore associations between maternal blood lipid status and infant adiposity.

**Results:**

All maternal blood lipids increased significantly during pregnancy. Maternal dietary fat intake was positively associated with total cholesterol levels in early pregnancy. Late pregnancy triglycerides were positively associated with birth weight (*P* = 0.03) while cord blood triglycerides were negatively associated with birth weight (*P* = 0.01). Cord HDL-C was negatively associated with infant weight at 6 months (*P* = 0.005). No other maternal blood lipids were associated with infant weight or adiposity up to 2 years of age.

**Conclusion:**

Maternal and fetal triglycerides were associated with birth weight and cord HDL-C with weight at 6 months. Thus, maternal lipid concentrations may exert *in-utero* influences on infant body composition. There may be potential to modulate infant body composition through alteration of maternal diet during pregnancy.

## Introduction

Obesity in childhood has become prevalent worldwide with the World Health Organisation estimating that over 41 million children aged under 5 years are obese [1]. Obese children are at increased risk of adult obesity and are at an increased risk of developing type 2 diabetes, cardiovascular disease and metabolic syndrome [2]. With such high levels of childhood obesity, it is vital to understand the variables associated with it, particularly the associations of maternal diet during pregnancy on the child’s body composition. As maternal diet during pregnancy is modifiable, this represents a way to potentially reduce childhood obesity levels.

Maternal nutrition impacts both the mother’s own health and can have a lasting impact on the health of the baby [3]. During pregnancy, the fetus is in a critical period of plasticity where it is influenced and moulded by the in-utero environment. During this time, fetal programming and the maternal environment influence the child’s health and development from birth up to adulthood [4–6]. The cellular mechanisms whereby the *in-utero* environment affects fetal development remain largely unknown. There is significant potential for the role of the maternal diet during this time, however with regard to specific macronutrients such as lipids, a dearth of knowledge remains. It is important to identify how specific elements of maternal nutrition interact with the child at birth and in later life to discover the dietary intakes that are associated with the best outcomes for both the mother and child.

It is estimated that glucose accounts for up to 80% of the energy used by the fetus during pregnancy and is the primary fuel for fetal growth [7]. However, information regarding the other 20% remains scarce. As research indicates that lipids can cross the placenta [8]; they could be responsible for an additional energy source to be used by the fetus. Maternal lipid concentrations also hold potential to influence fetal lipid concentrations [7]. Research examining the interaction between maternal cholesterol and fetal development found that in the first trimester up to 20% of cholesterol accumulated by the developing fetus was obtained from the mother’s own cholesterol stores [7]. But a paucity of research remains in relation to the impact of high concentrations of maternal cholesterol on the developing fetus.

For non-pregnant individuals, the recommendations for healthy concentrations of blood lipids are; below 5mmol/L for total cholesterol, below 3mmol/L for low-density lipoprotein cholesterol (LDL-C), below 2mmol/L for triglycerides and above 1mmol/L for high-density lipoprotein cholesterol (HDL-C) [9]. Although these blood lipids all naturally increased during pregnancy [10–13], they are not routinely measured as part of antenatal care. Some studies have published their own values for small reference groups [10,13,14] however currently there are no guidelines for normal cholesterol or triglyceride concentrations during pregnancy.

There is a scarcity of data on the association between blood lipids during pregnancy and infant outcomes, with the limited knowledge focusing on women with gestational diabetes mellitus (GDM). Triglyceride concentrations in women with hyperglycaemia during pregnancy have been shown to be associated with increased birth weight [15,16]. While some studies have been performed addressing the association between maternal blood lipid profiles in euglycaemic women and infant size [17,18], conflicting reports have been found in this understudied area. We therefore aimed to further elucidate the relationship between mother’s blood lipid concentrations and fetal and infant anthropometry in a euglycemic cohort.

## Materials and Methods

### Study Design

Analysis was carried out on a subset of 331 mother and child pairs from the ROLO (Randomised cOntrol trial of LOw glycaemic index diet vs no dietary intervention in pregnancy to prevent recurrence of a large baby) study. This randomised controlled trial (registration number ISRCTN54392969) was carried out in The National Maternity Hospital, Dublin, Ireland. Ethical approval was granted by the National Maternity Hospital Ethics Committee and maternal written consent was obtained. Eight hundred secundigravida women who did not have gestational diabetes but had previously given birth to a macrosomic baby (birth weight equal to or above 4.0 kg), and were therefore at increased risk of delivering another macrosomic infant [19,20], were randomised to receive low glycaemic index (GI) dietary advice or usual antenatal care, which did not include dietary advice. Detailed methods [20] and dietary results [21,22] of the ROLO study have previously been published In brief; the low GI diet resulted in lower dietary glycaemic load, but did not impact on birth weight [20]. However significant maternal benefits were noted in terms of less gestational weight gain (12.2 kg vs 13.7 kg, *P* < 0.05) and less glucose intolerance, which was diagnosed using a glucose challenge test measuring serum glucose above 7.8mmol/L (21% vs 28%, *P* < 0.05) [20].

### Data Collection

All participants had their height and weight measured by trained healthcare professionals at the first 14-week antenatal visit and body mass index (BMI kg/m^2^) was calculated. Additional demographic information collected included mothers’ education level and smoking status. Three-day food diaries were completed during each trimester to estimate dietary intake throughout the pregnancy. Dietary fat intake was calculated for each trimester using nutritional analysis software NetWISP version 3.0 (Tinuviel software, Llanfechell, Anglesey, UK) which utilises the food composition database from the 6^th^ edition of McCance and Widdowson’s food composition tables.

Infants were measured at birth, 6 months and 2 years of age for weight and recumbent length along with abdominal circumference and bicep, tricep, subscapular and thigh skinfold thicknesses. From these measurements, weight centiles, waist:length ratio, subscapular:tricep ratio, sum of skinfolds and sum of tricep and subscapular skinfolds were calculated, which were used as measures of neonatal adiposity. At the 6-month and 2-year follow-up appointments, mothers gave details on breastfeeding habits and duration.

### Laboratory Analyses

Maternal fasting blood samples were taken in early pregnancy (approximately 14 weeks’ gestation), late pregnancy (28 weeks’ gestation) and a cord blood serum sample was collected at delivery. Serum total cholesterol, HDL cholesterol and triglyceride concentrations were measured using Roche cholesterol oxidase method, lipase/GPO-PAP (glycerol phosphate oxidase-p-aminophenazone) and direct HDL-Roche 3rd generation method respectively, on the cobas c702 module of the Roche Cobas 8000 analyser in accordance with the manufacturer’s instructions (Roche Diagnostics GmbH, Penzburg, Germany). The methods are standardised against the isotope dilution/mass spectrometry (ID/MS) methods. LDL-cholesterol concentration was estimated using the Friedewald equation [23]. Multianalyte profiling was performed on the Luminex Mag-pix system (Luminex Corporation, Austin, Texas) in accordance with the manufacturer’s instructions. The Human Endocrine Panel was used to determine blood plasma concentrations of insulin and leptin (in early pregnancy, late pregnancy and cord samples) and C-peptide (in the cord serum sample). Maternal insulin resistance was calculated using the homeostatic model assessment (HOMA) index: HOMA score = (fasting insulin μU/mL x fasting glucose mmol/L)/22.5 [24]. The techniques and the laboratory that carried out these analyses were calibrated to National Institute of Standards in Technology reference materials.

### Statistical Analyses

Membership of the control or intervention group of the ROLO study had no association with blood lipid levels or infant anthropometry so both groups were analysed together. All variables were assessed for normality using the Shapiro-Wilk test and by visual analysis of histograms. Relationships between the central tendencies were examined using paired sample *t*-tests. For parametric data, Pearson correlation was used, and Spearman’s correlation for the non-parametric data to individually measure the correlation between each blood lipid (in early and late pregnancy and cord blood), HOMA, C-peptide and leptin concentration and each of the anthropometric measures of child weight and adiposity (at birth, 6 months and 2 years of age). Bivariate associations at a significance of *P* < 0.1 were considered significant and were further analysed using multiple regression models. Multiple regression models were created separately for each outcome of child weight or adiposity using a forced entry approach to include the following confounders; at birth: mother’s BMI at the first visit, infant gestational age at birth, infant gender, mother’s education status, and mother’s smoking status, and at 6-month and 2-years: gender, age at data collection, mother’s education status and whether the child was breastfed. The final multiple linear regression models that were statistically significant (*P* < 0.05) were reported as the best predictors of infant weight and adiposity. All statistical analyses were carried out using SPSS (Statistical Package for the Social Sciences) software version 20.0 (IBM, Armonk, NY).

## Results

### Characteristics of the Cohort

Mean BMI at 14 weeks’ gestation was 26.4 kg/m², which is in the overweight category (**Table 1**). Just over half (54.4%) of the study participants had a BMI over 25 kg/m². Mean infant birth weight was 4.076 kg with 54.7% of infants classified as macrosomic or large-for-gestational-age. Mean birth weight centile was 72.9. Average infant weight was 8.4 kg at 6 months and 13.1 kg at 2 years. Mean maternal daily intake of saturated fat was 72.4g in trimester 1 and 73.0g in trimester 2, mono-unsaturated fat was 28.1g in trimester 1 and 28.4g in trimester 2 then poly-unsaturated fat intakes were 22.9g and 23.1g in trimester 1 and 2 respectively.

**Table 1:** Characteristics of mothers and infants.

Characteristics	Mean (SD)
**Maternal Characteristics**	
**Age at delivery (years)**	33.10 (3.90)
**BMI at 14 weeks’ gestation (kg/m²)**	26.40 (4.60)
**Achieved 3**^**rd**^ **level education (n(%))**	174 (58.6)
**Smoked during pregnancy (n(%))**	7 (2.1)
**Neonatal/Infant Characteristics**	
**Male gender (n(%))**	157 (47.4)
**Gestational age at delivery (days)**	282.80 (7.50)
**Ever breastfed (n(%))**	103 (64)
**Birth weight (kg)**	4.07 (0.47)
**Birth weight centile**	72.90 (24.90)
**Birth waist:length ratio**	0.64 (0.04)
**Birth sum of skinfolds (mm)**	28.40 (5.30)
**Birth subscapular:tricep skinfold ratio**	1.01 (0.20)
**6-month weight (kg)**	8.44 (1.30)
**6-month weight centile**	69.45 (24.79)
**6-month waist:length ratio**	0.64 (0.03)
**6-month sum of skinfolds (mm)**	41.62 (4.81)
**6-months subscapular:tricep skinfold ratio**	1.97 (0.48)
**2-year weight (kg)**	13.07 (1.74)
**2-year weight centile**	68.09 (25.86)
**2-year BMI (kg/m²)**	16.39 (1.54)
**2-year BMI centile**	60.30 (28.30)
**2-year waist:length ratio**	0.56 (0.06)
**2-year sum of skinfolds (mm)**	41.02 (5.92)
**2-year subscapular:tricep skinfold ratio**	2.25 (0.73)

BMI: Body mass index

All data expressed as either mean (SD) or n (%)

### Blood Lipid Concentrations in Pregnancy

Concentrations of total cholesterol, non-HDL cholesterol, HDL-C, LDL-C and triglycerides increased significantly from early pregnancy to late pregnancy (**Table 2**).

**Table 2:** Concentrations of blood lipids (Median (IQR).

Timepoint	Total Cholesterol	Non-HDL Cholesterol	HDL-C	LDL-C	TG
**Early Pregnancy (n = 284)**	4.58 (3.87–5.39)	3.85 (3.15–4.53)	0.64 (0.46–0.97)	3.31 (2.66–3.94)	1.31 (0.80–1.35)
**Late Pregnancy (n = 293)**	6.02 (5.00–6.87)*	5.02 (4.01–5.98)*	0.85 (0.54–1.13)*	4.15 (3.43–5.06)*	1.71 (1.28–2.19)*
**Cord Blood (n = 228)**	1.70 (1.43–1.99)	1.24 (1.00–1.42)	0.49 (0.36–0.61)	0.99 (0.79–1.21)	0.46 (0.35–0.60)

Results displayed in mmol/L, HDL-C: High density lipoprotein cholesterol, IQR: Inter-quartile Range, LDL-C: Low density lipoprotein cholesterol, TG: Triglycerides.

* denotes a significant increase from early to late pregnancy (*P* < 0.001)

### Dietary Fat and Blood Lipids

Dietary saturated fat intake in trimester one was positively associated with total cholesterol in early pregnancy (*P* = 0.02). Likewise, dietary intakes of monounsaturated and polyunsaturated fat were positively associated with total cholesterol at this timepoint (*P* = 0.005 and *P* = 0.038, respectively). Dietary fat intake was not associated with triglyceride concentrations in early or late pregnancy, however, there was a negative association between intakes of saturated, monounsaturated and polyunsaturated fat in trimester two and triglyceride concentrations in cord blood (*P* = 0.011, *P* = 0.011 and *P* = 0.013 respectively).

### Bivariate Associations

Birth weight was associated with triglycerides in late pregnancy and triglycerides and LDL-C in cord blood. At 6 months, infant weight was associated with total cholesterol and HDL-C in cord blood while weight centile was associated with total cholesterol in the cord. Six-month waist:length ratio was associated with total cholesterol and LDL-C in cord blood, and sum of skinfolds was associated with triglyceride concentrations in early and late pregnancy, HDL-C in late pregnancy and LDL-C in cord blood. Subscapular:tricep skinfold ratio was associated with HDL-C in late pregnancy and total cholesterol, LDL-C and triglycerides in the cord blood. At 2 years, weight was associated with total cholesterol and LDL-C in cord blood. 2-year weight centile was associated with total cholesterol at all three time points, non-HDL cholesterol and LDL-C and triglycerides in late pregnancy and cord blood. Two-year old waist:length ratio was associated with HDL-C in both early and late pregnancy and cord blood, sum of skinfolds was associated with LDL-C in early pregnancy and triglyceride concentrations in the cord. Two-year subscapular:tricep ratio was associated with HDL-C in early and late pregnancy. No other blood lipid concentrations in early pregnancy, late pregnancy or cord blood were associated with neonatal and infant anthropometry or skinfold thicknesses. No associations were found between HOMA or C-peptide and child weight and adiposity.

### Multiple Regression Models

After controlling for confounders (at birth: mother’s BMI, gestational age, infant gender, mother’s education and smoking status, and at 6-month and 2-years: infant gender, age at data collection, mother’s education status and breastfeeding), outcomes associated with maternal blood parameters were birth weight, birth weight centile, and weight at 6 months. Birth weight was significantly associated with triglyceride concentrations in late pregnancy and cord as well as leptin concentrations in the cord (*P* = 0.03, *P =* 0.01 and *P =* 0.02, respectively) (**Table 3**). Late pregnancy triglycerides were positively associated with birth weight (*B* = 111.17) while cord blood triglycerides were negatively associated with birth weight (*B* = -453.75). Based on the adjusted r² value, this model explains 31.8% of the variation in birth weight. The same variables were significant in the model for the outcome of birth weight centile (data not shown). There was no association with HOMA (measured in early or late pregnancy) with triglyceride level however late-pregnancy triglycerides were correlated with cord C-Peptide levels (*P* = 0.001). When C-Peptide was added to the model as a confounder the association of triglycerides in late pregnancy with birth weight was weakened (*P* = 0.059).

**Table 3:** Multiple regression model examining the associations between maternal blood lipids and birth weight as an outcome.

Model	*B*	*P*	CI Lower	CI Upper	r² adj	F	*P*
**TG (late)**	111.17	0.034	8.48	213.87	0.318	6.54	<0.001
**TG (cord)**	-453.75	0.013	-811.77	-95.73			
**LDL-C (cord)**	88.55	0.426	-131.21	308.3			

Outcome = Birth weight. TG: Triglycerides, LDL-C: Low density lipoprotein-cholesterol.

Controlled for mother’s BMI, education level, smoking during pregnancy, infant gestational age and gender and leptin in early pregnancy and cord blood.

**Fig 1:**
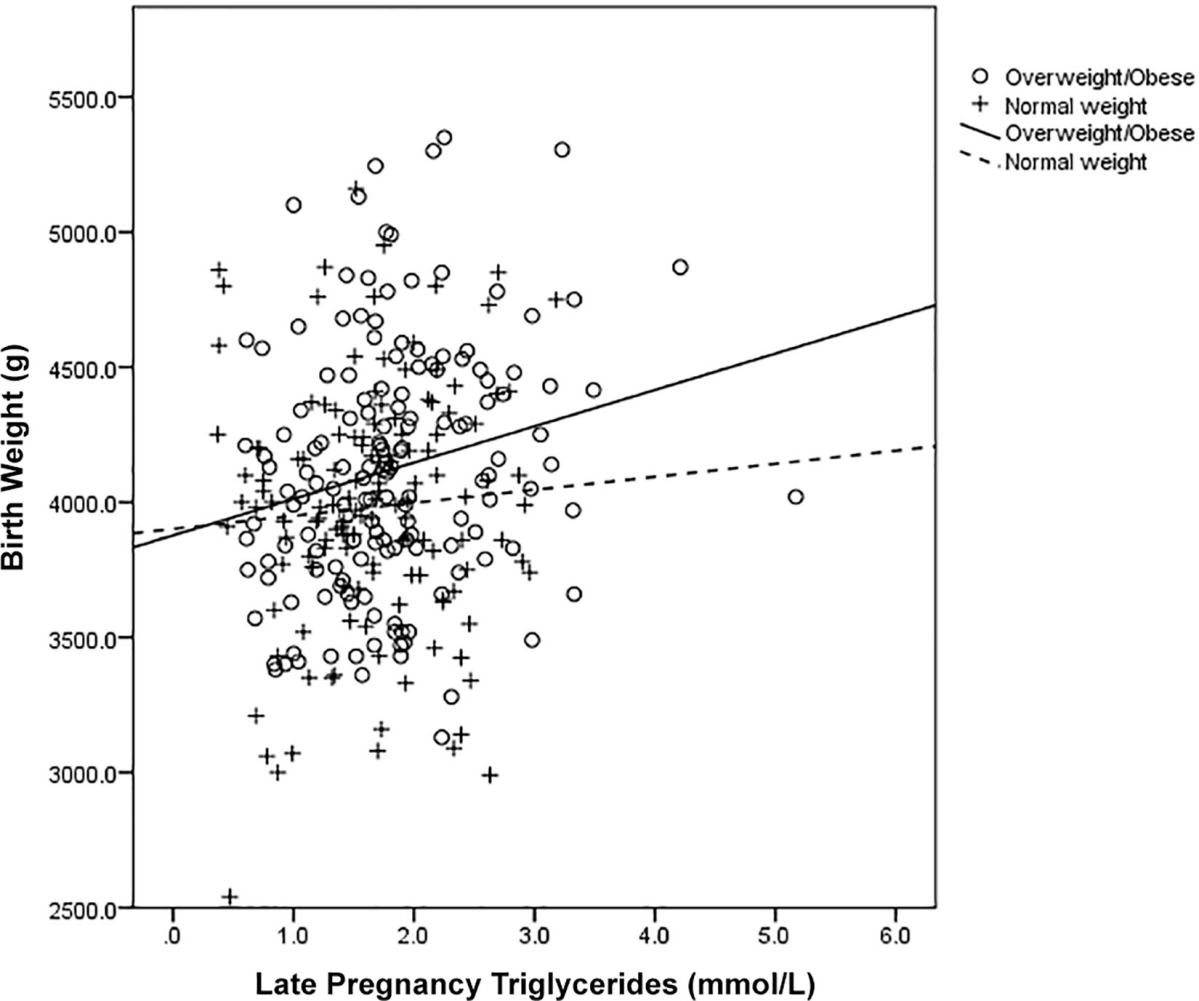
Scatter plot displaying the association between birth weight and late pregnancy triglyceride concentrations stratified by BMI category.

**Fig 2:**
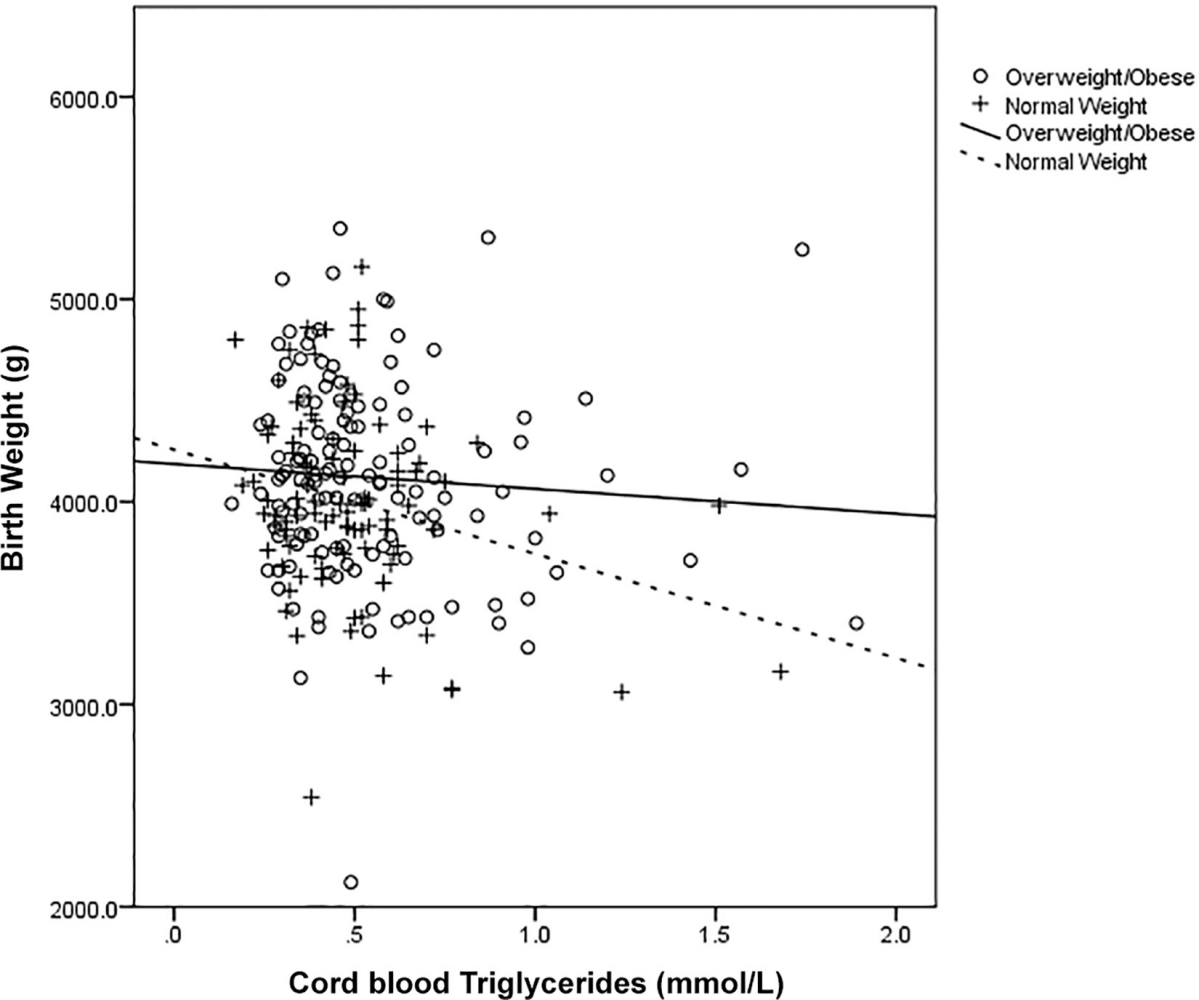
Scatter plot displaying the association between birth weight and cord triglyceride concentrations stratified by BMI category.

When maternal fasting glucose concentrations (in early and late pregnancy) were added into the model as additional confounders for birth weight this attenuated the association of triglycerides in late pregnancy (P = 0.07) however triglycerides in cord blood remained significantly associated (P = 0.02). Upon stratification of these data by maternal BMI at 14 weeks’ gestation, we found that the triglyceride associations differed in overweight or obese mothers compared to normal-weight mothers (**Fig 1** and **Fig 2**). When the adjusted regression analysis was conducted separately among normal-weight mothers (BMI <25 kg/m^2^) and overweight/obese mothers (BMI ≥25 kg/m^2^), late pregnancy triglycerides were associated with birth weight in overweight and obese mothers only (R² = 0.08 and P = 0.008 versus R² = 0.0003 P = 0.92 in BMI <25 kg/m²) and cord triglycerides were associated with birth weight in mothers with a BMI below 25 only (R² = 0.08 and P = 0.01 versus R² = 0.001 and P = 0.69 in BMI ≥25 kg/m²).

The final multivariate model and third infant outcome that was significantly associated with maternal blood parameters was weight at six months (**Table 4**). HDL-C concentration from cord blood was negatively associated with weight at 6 months (*P =* 0.005) and along with gender, child age, mother’s education level and breastfeeding, this model explained 18.8% of the variance in weight at 6 months (based on the adjusted r² value). In the final models, no other maternal blood lipids were associated with weight or adiposity up to two years of age.

**Table 4:** Multiple regression model examining the associations between maternal blood lipids and 6-month infant weight as an outcome.

Model	*B*	P	CI Lower	CI Upper	r² adj	F	P
**HDL-C (cord)**	-2.462	0.005	-4.14	-0.79	0.188	4.06	0.003

Outcome = weight at 6 months. HDL: High density lipoproteins. Controlled for child’s gender, age at data collection, mother’s education level, breastfeeding.

## Discussion

In this cohort, the concentrations of all blood lipids increased significantly as pregnancy progressed. Striking results were found regarding maternal triglyceride concentrations and birth weight. Late pregnancy triglyceride concentrations were positively associated with birth weight, while triglyceride concentrations in cord blood were negatively associated. Leptin concentrations in cord blood were positively associated with birth weight, as expected. Leptin is an adipokine secreted from adipose tissue and concentrations rise significantly with increasing percentage of body fat [25].

Part of the normal physiological changes that occur in pregnancy involve a large increase in the mother’s blood volume by approximately 45% [26]. The present data indicate that blood lipid concentrations also increase in line with this. The increase in serum lipids during pregnancy has been shown in previous research [10–13] with one group of researchers finding that a mother’s serum cholesterol concentrations rise by approximately 50–70% during pregnancy compared to normal concentrations [27]. Others have reported that, compared to non-pregnant women, estimated total cholesterol concentrations increase up to 39% in late pregnancy and triglyceride concentrations may be anywhere up to 138% higher than non-pregnant concentrations in late pregnancy [12]. Evidence has shown that blood lipids revert to pre-pregnancy levels after delivery which suggests that the elevated serum lipids could play an important role in fetal development [12,13,27].

It is hypothesised that triglycerides may be used by the developing fetus as a fuel for growth, in addition to glucose [28]. The higher concentrations of triglycerides available in the mother’s blood during pregnancy could allow the fetus to develop a larger placenta [29]. Research has shown that the placental capacity for nutrient transfer increases between mid and late gestation with placental surface area for nutrient exchange estimated to increase 5–15 fold [29]. The results obtained for the late pregnancy triglyceride concentrations are in line with previous research that found associations between high triglycerides during pregnancy and increased birth weight [15,17,28,30]. A study carried out on under-nourished pregnant women found blood triglycerides and HDL-C to be as influential as glucose in determining birth weight [28]. Although their cohort was quite different to the current cohort in terms of ethnicity and maternal weight, similar associations were found. Two other studies also found that mid-pregnancy triglyceride concentrations were positively associated with and could independently predict birth weight [15,30].

Upon further analysis of this cohort, the associations between the triglyceride levels and birth weight differed by BMI. There was a positive association between late pregnancy triglycerides and birth weight among mothers with a BMI over 25 kg/m^2^. A possible mechanism could be that these overweight or obese mothers have altered metabolism with increased insulin resistance levels and utilise triglycerides for their own energy production which increases the amount of glucose directly available to the fetus. This hypothesis is supported by our findings which showed that when glucose levels were controlled for, the association between triglycerides in late pregnancy and birth weight was attenuated. There was a positive association between late-pregnancy triglycerides and cord C-peptide, which research has previously shown to be an indicator of maternal insulin and metabolic functioning [31]. This idea is also supported by recent evidence from a study examining lipid levels in healthy pregnancies that found that due to increased insulin resistance mothers may be using lipids as an alternative energy source [32].

Research has suggested that an increase in placental lipoprotein lipase (LPL) activity facilitates greater fatty acid placental transfer and could contribute to higher fetal fat accumulation [8]. Animal studies have shown that maternal high fat intake has been linked to increased LPL in the placenta which increased triglyceride uptake [33]. Taking this into account, it is plausible that the placentae of women with a BMI under 25 kg/m^2^ may have less placental LPL, which could decrease the uptake of triglycerides by the placenta, which may in turn be associated with lower weight babies, as found in this study. Similar findings relating to the negative association of triglycerides in cord blood with birth weight have only been reported in one other study. Schaefer-Graf *et al*. also found that in a cohort of women with well-controlled GDM, high concentrations of cord blood triglycerides were associated with decreased birth weight [34]. They also hypothesised that low triglyceride concentrations found in the cord blood of large-for-gestational-age babies could be the result of enhanced placental lipoprotein lipase activity derived from their increased adipose mass [34]. Further research needs to be carried out in this area.

There is limited knowledge on the relationship between maternal HDL-C concentrations during pregnancy and fetal development and infant growth. While HDL-C is not thought to cross the placenta, it has been shown in animal studies to influence cholesterol metabolism in the placenta which could affect the size of the fetus as a result [35]. We found that higher HDL-C concentrations in cord blood were associated with lower infant weight at 6 months. To our knowledge this is the first study to examine the associations between HDL-C concentrations in pregnancy and cord blood and infant weight at 6 months of age. A recently published study reported a negative association between HDL-C concentrations measured near the end of pregnancy and birth weight [36]. The authors reported that lower HDL-C concentrations were a predictor for large-for-gestational age babies [36]. Similarly, another study reported that higher HDL-C concentrations in late pregnancy were associated with small-for-gestational-age babies [37]. With this in mind, while we found no direct association on birth weight, it is possible that higher HDL-C concentrations can result in lower weight babies that remain small as they grow, with our findings suggesting that this can have a lasting impact up to 6 months of age.

Dietary intakes of fat in early pregnancy were associated with total maternal cholesterol concentrations. Previous research in non-pregnant cohorts has shown that an individual’s intake of fatty acids influences their cholesterol concentrations [38] and animal studies have indicated that a high fat diet during pregnancy can modulate the offspring’s blood lipid profile in later life [39]. Previous research on the ROLO cohort has also linked dietary fat intake with neonatal adiposity [22], where saturated fat intake was positively associated with central adiposity. In our analysis, saturated fat was associated with reduced triglycerides in cord blood which was associated with higher birth weight. With LPL expression also being associated with dietary fat intake [33] this could potentially be an additional mechanism by which maternal diet can influence the offspring anthropometry. Further research is needed to elucidate this complex relationship.

This study adds to the current knowledge of associations between maternal blood lipid concentrations during pregnancy and outcomes for the baby. A comprehensive meta-analysis found that women with gestational diabetes have elevated triglyceride concentrations [40], and research shows that lifestyle factors during pregnancy can also influence maternal triglyceride concentrations [41]. With this in mind, it may be prudent to monitor serum lipids closely throughout pregnancy alongside the routine checking of glucose concentrations. It is also advisable to establish healthy reference intervals for blood lipids during pregnancy. The findings presented here add more evidence to the literature that suggests that the fetus is not solely dependent on glucose for growth. It is vital that research is carried out examining the impact of different nutrients on the fetus. As blood lipid concentrations could potentially be modulated by maternal diet, this opens opportunities for dietary interventions during pregnancy and monitoring cholesterol and triglyceride concentrations closely to ensure a healthy-weight baby.

This study had many strengths in that it was one of few longitudinal studies to have maternal and fetal blood samples at two time points during pregnancy, along with detailed infant anthropometry up to 2 years of age. These data allowed us to investigate the complex relationship between blood markers at each time point and fetal growth in later life. All anthropometric measurements (including maternal BMI) were collected by trained healthcare professionals rather than based on self-reports. As with all studies there were some limitations. While there was a reasonably large sample size and the cohort was healthy, average maternal BMI was in the overweight category. In addition, these mothers had previously given birth to a macrosomic infant and the offspring were, on average, in the macrosomic range. Analysis was carried out and there was no association between maternal lipid profiles and rates of macrosomia but, that being said, the results presented may not be representative of all pregnant and infant populations.

## Conclusions

In the present cohort, maternal blood lipid concentrations in late pregnancy and in cord blood were associated with offspring anthropometry. Maternal triglyceride concentrations were associated with birth weight and HDL-C in cord blood was negatively associated with infant weight at 6 months of age. These results suggest that maternal lipid concentrations may exert an *in-utero* influence on later infant body composition, and this could be modulated by maternal BMI. Further mechanistic research is required to elucidate the complex relationship between maternal blood lipid concentrations and fetal growth and development. There is a need to establish recommended healthy blood lipid concentrations during pregnancy. With dietary intakes of fat during pregnancy potentially influencing maternal blood lipid profiles, there is the potential to modulate infant body composition by altering the mother’s diet during pregnancy.
